# Combining biocatalytic and radical retrosynthesis for efficient chemoenzymatic synthesis of natural products

**DOI:** 10.1039/d5cs00453e

**Published:** 2025-07-22

**Authors:** Hans Renata

**Affiliations:** a Department of Chemistry, Rice University, BioScience Research Collaborative Houston TX 77005 USA hrenata@rice.edu

## Abstract

Retrosynthetic strategies are intimately tied to the tools and methods available at the time of their conception. Recent methodological advancements in both radical and biocatalytic reactions have created numerous possibilities for new and unconventional disconnections. Thus, it comes as no surprise that their applications in multi-step syntheses have allowed many complex molecules to be built in ingenious ways. This review summarizes recent case studies wherein radical-based and enzymatic transformations are combined strategically in multi-step sequences to achieve efficient total syntheses of complex natural products.

## Abstract 2

This review highlights recent case studies of natural product synthesis wherein enzymatic and radical-based transformations are strategically used in combination to achieve efficient synthesis.

## Graphical abstract

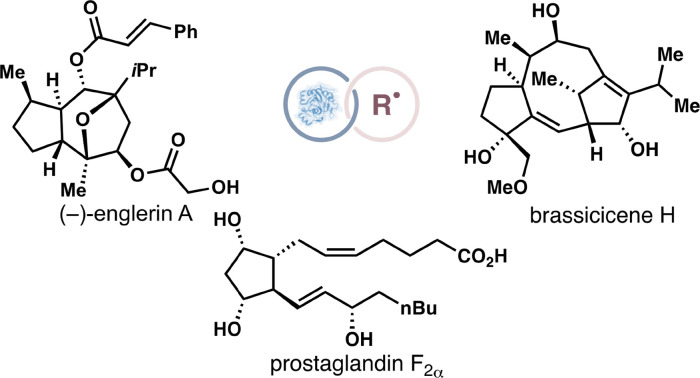

## 1. Introduction

The one-electron nature of radical reactions offers unique modes of reactivity for building complex molecules that are otherwise unavailable with two-electron processes. Spurred by the invention of new technologies in photoredox catalysis,^1^ modern electrochemistry,^2^ metal-catalyzed cross-coupling^3^ and hydrogen atom transfer^4^—which offer milder reaction conditions and better functional group compatibility than “classical” radical reactions—the field of radical chemistry has witnessed a major renaissance in recent years.^5^ In addition to these advantages, modern radical chemistry also allows for the use of unconventional functional groups such as carboxylic acids^6^ and alkenes^4^ as viable radical precursors, opening the door for profoundly different bond disconnections during synthesis planning. In the same vein, biocatalytic retrosynthesis^7^ has recently emerged as an enabling paradigm in chemical synthesis, owing to the unique selectivity profile of enzymatic reactions and the ever-increasing ability to modulate the activity and selectivity of enzymes with directed evolution and protein engineering. The transformative effects of this paradigm are manifest in the recent discoveries of new enzymes for site-selective oxidations,^8^ the advent of new-to-nature biocatalytic reactions^9^ and several high-profile reports^10^ on the use of enzymatic cascades in the preparation of drug molecules and active pharmaceutical ingredients.

Recently, various research groups have managed to capitalize on the unique features of biocatalytic and radical retrosynthetic logic and strategically combine them to achieve efficient chemoenzymatic synthesis of complex natural products. Generally speaking, these cases can be divided into two categories: (1) using enzymatic cyclization to construct the core architecture of the target natural product, followed by radical-based chemical reactions to functionalize the core, or (2) using radical-based C–C bond formations to generate the core architecture of the target natural product, which is done in combination with enzymatic tailoring, usually oxidation, to install the requisite functional groups. Coincidentally, it should be noted there has been a heavy emphasis on applying these two chemoenzymatic approaches to terpenoid natural products. This review will be organized according to the two aforementioned categories. Additionally, we will present at the end several examples that involve non-terpenoid targets such as fatty acids and alkaloids.

## 2. Enzymatic cyclization followed by radical functionalization – introduction to terpene cyclases

Terpenoids are chemically and structurally diverse hydrocarbon-based natural products that arise from two 5-carbon precursors, isopentenyl diphosphate (IPP) and dimethylallyl diphosphate (DMAPP).^11^ Initial head-to-tail coupling of IPP and DMAPP yields linear, achiral C_5*n*_ isoprenoid diphosphates (*n* = 1, 2, 3, *etc.*), which undergo various modes of cyclization by terpene cyclases to deliver products interesting three-dimensional architectures. Among the terpene cyclases,^12^ class I terpenoid cyclases, such as bacterial pentalenene synthase, utilize a trinuclear metal cluster to activate pyrophosphorylated substrates, whereas class II terpenoid cyclases, such as SHCs, employ an active site side chain to protonate alkenes or epoxides for cyclization initiation.

Nature uses two pathways to generate the isoprenoid diphosphate precursors: the mevalonate (MVA) pathway^13^ and (ii) the methylerythritol phosphate (MEP) pathway.^14^ The mevalonate pathway begins with the conversion of acetyl CoA to acetoacetyl CoA, followed by an aldol coupling with another acetyl CoA molecule to generate (3*S*)-3-hydroxy-3-methylglutaryl CoA. Subsequent reduction affords (*R*)-mevalonate, which undergoes a series of reactions to provide isopentenyl diphosphate (IPP). The MEP pathway, sometimes referred to as the deoxyxylulose phosphate (DXP) pathway was only discovered in 1993, and is less well-studied relative to the MVA counterpart. The pathway starts with the union of pyruvate and d-glyceraldehyde-3-phosphate to produce DXP, which undergoes rearrangement in the presence of DXP reductase to provide 2*C*-methyl-d-erythritol-4-phosphate. Phosphorylation of this intermediate and reduction forms IPP. Finally, isomerization of IPP to DMAPP takes place in the presence of IPP isomerase, which becomes the starting point for further oligomerization to generate longer C_5*n*_ cyclization precursors, such as geranyl diphosphate (C10), farnesyl diphosphate (C15) and geranylgeranyl diphosphate (C20).

Several chemoenzymatic approaches to complex terpenoids featuring the use of terpene cyclases have recently been reported. The overall strategy is to employ a terpene cyclase for initial construction of the carbocyclic skeleton, followed by chemical tailorings to deliver the final terpenoid target(s). From a step economy perspective, the advantage here is obvious as the desired carbocyclic skeleton can be obtained in just one step. However, many of these enzymatic pathways, especially when carried out *in vivo*, are not efficient enough to generate sufficient materials to support a multi-step synthesis campaign. For this reason, it is often necessary to perform additional metabolic engineering on the bacterial and fungal host, typically to enhance precursor supply for the enzymatic cyclization. To date, engineering of the MVA pathway has been more widely explored for this purpose than that of the MEP/DXP pathway. In recent years, several groups have taken advantage of the ability to readily access various terpene skeletons with terpene cyclases and used it in combination with modern radical reactions for efficient chemoenzymatic syntheses of complex terpenoids.

### 2.1. Chemoenzymatic synthesis of artemisinin

First isolated in 1972, the sesquiterpene artemisinin (1) is a widely-used small molecule for the treatment of malaria.^15^ In fact, artemisinin-based combination therapies (ACTs) are now the standard treatment worldwide for malaria caused by *Plasmodium* species. The semi-synthetic artemisinin project was established in 2014 to improve the supply of artemisinin and lower its production cost through a two-stage approach involving microbial production of artemisinic acid (2), followed by its chemical conversion to artemisinin.^16^ This project was highly successful and is often cited as an important milestone in metabolic engineering in the past two decades.

**Fig. 1:**
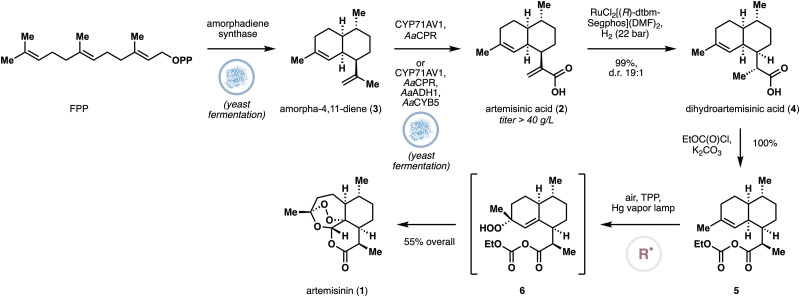
Semisynthetic production of artemisinin through heterologous production of artemisinic acid and its chemical derivatization.

In 2006,^17^ Keasling and coworkers engineered a *S. cerevisiae* strain that is equipped with an engineered MVA pathway, an amorphadiene synthase, and the P450 CYP71AV1 from *A. annua* to produce 2 with a titer of 100 mg L^−1^ (Fig. 1). In this pathway, amorpha-4,11-diene (3) is produced by the synthase and gets converted by the P450 to 2. Subsequent work by Paddon and coworkers^18^ successfully doubled the titer production of 2 by overexpressing every enzyme in the MVA pathway up to ERG20 in an engineered *S. cerevisiae*. This achievement was complemented by optimization of the fermentation process to improve the titer further to >40 g L^−1^. In 2013,^19^ Newman and coworkers reported that optimization of the oxidation of 3 to 2 could lead to a high-level production of 2 (25 g L^−1^) by yeast fermentation. Specifically, the researchers lowered the expression of *Aa*CPR, which presumably affected the stoichiometry of CYP71AV1:*Aa*CPR interaction, and introduced several auxiliary proteins such as cytochrome B5. Prior work in P450 biochemistry has suggested that the P450:CPR stoichiometry is important for optimal oxidation efficiency. Conversion of artemisinic acid to artemisinin has been widely studied and has even been conducted on process scale.^20^ In the sequence, the *exo* methylene is first reduced, and the acid is converted to either the ester or the mixed anhydride (*e.g.*, 5), which was then subjected to a Schenck ene/rearrangement cascade with ^1^O_2_—which has been proposed to proceed *via* radical mechanism—to furnish artemisinin. This last step has been studied extensively on process scale due to the need to develop special photochemical setups. For example, Sanofi has introduced a semibatch process with a recirculation loop while carefully choosing the reactor materials the identity of the photon source for optimal quantum photonic yield.

### 2.2. Chemoenzymatic synthesis of englerin A

**Fig. 2:**
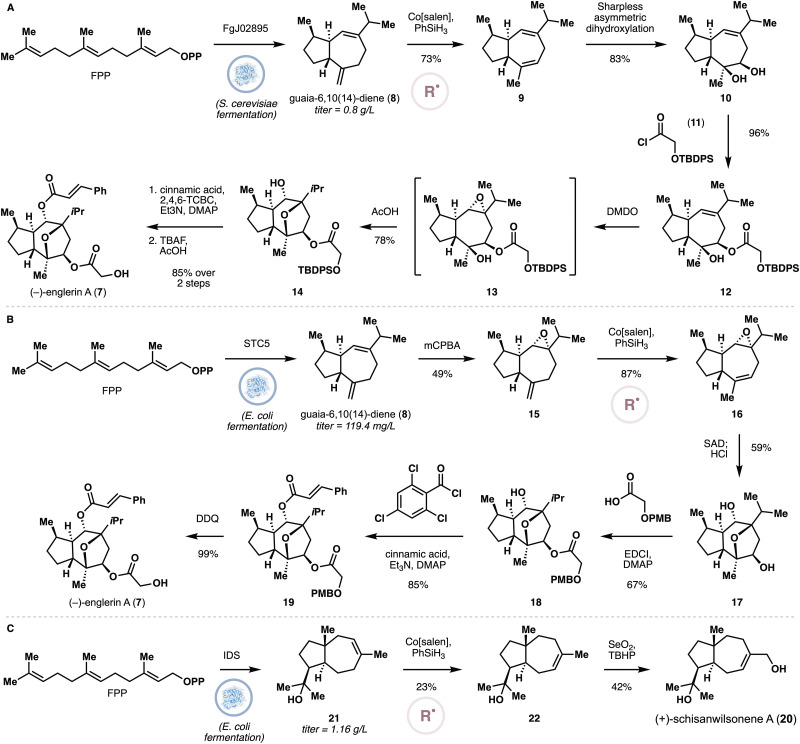
(A) Chemoenzymatic synthesis of englerin A by Liu, Christmann and coworkers. (B) Chemoenzymatic synthesis of englerin A by Xiang and coworkers. (C) Xiang's chemoenzymatic synthesis of schisanwilsonene A.

Englerin A (7) is a plant sesquiterpenoid with potent nanomolar cytotoxicity against renal cancer cells,^21^ which was later rationalized by its ability to activate the calcium channels TRPC4 and TRPC5.^22^ In light of its potent bioactivity and unusual structure, more than 20 total and formal syntheses of 7 have been reported to date. In 2020, Liu, Christmann and coworkers^23^ develop a concise synthesis of 7 by relying on the heterologous production of guaia-6,10(14)-diene (8) in *S. cerevisiae*, followed by further chemical manipulations (Fig. 2A). Christmann, Dickschat and coworkers previously discovered STC5^24^ as a class II sesquiterpene cyclase that converts FPP to 8. Due to the low isolation yields (4%) of the system, they tested additional sesquiterpene cyclases from filamentous fungi for guaia-6,10(14)-diene production in an *E. coli* strain that has been manipulated to overproduce FPP. The screening resulted in a slightly improved production of 8 at up to 62.3 mg L^−1^ with *E. coli* mutant G5, which contains the cyclase FgJ02895. Aiming at a more practical production, they further engineered the enzymes in the MVA pathway by CRISPR/Cas9 system in *S. cerevisiae*, and the titer of 8 was further increased to 0.8 g L^−1^ in 5 L fed-batch fermentation of *S. cerevisiae* YL06. Under Shenvi's conditions for hydrogen atom transfer-based olefin isomerization,^25^8 was converted to diene 9, which was then subjected to a regio- and diastereoselective Sharpless dihydroxylation. The secondary alcohol was selectively acylated in the presence of acid chloride 11 and the remaining alkene was epoxidized with DMDO. *In situ* treatment of the epoxide with AcOH induced further cyclization to provide the core scaffold of englerin A. Introduction of the cinnamyl ester and removal of the TBDPS group completed the semisynthesis of 7.

### 2.3. Chemoenzymatic synthesis of schisanwilsonene A

The Xiang group has also developed an alternative approach (Fig. 2B) to heterologously produce guaia-6,10(14)-diene (8),^26^ which used *E. coli* as the heterologous host. Specifically, the researchers employed a two-plasmid system wherein the MVA pathway was incorporated in the vector pACYCDuet-T1-B1 while the FPP synthase (FPPS) ERG20 and the sesquiterpene synthase STC5 were encoded in the vector pETDuet-ERG20-STC5. This system delivered 8 with a titer of 119.4 mg L^−1^. While the terpene starting point is identical to that of Liu *et al.*, the Xiang group developed an alternative sequence to convert it to 7. Firstly, *m*CPBA was used to selectively epoxidize the trisubstituted olefin. *Exo*-to-*endo* isomerization of the remaining alkene was achieved under MHAT-based conditions, which was followed by dihydroxylation and transannular cyclization in the presence of HCl to afford 17. Two sequential esterifications and deprotection of the PMB protecting group completed their synthesis of englerin A.

Using a similar heterologous expression system, the Xiang group has also reported a two-stage synthesis of (+)-schisanwilsonene A (20, Fig. 2C).^27^ Firstly, introduction of the terpene cyclase IDS^28^ in place of STC5 led to the accumulation of (+)-isodauc-8-en-11-ol (21) with a titer of 1.16 g L^−1^. Under MHAT conditions for alkene isomerization, 21 was converted to 22 in 23% yield. Allylic oxidation of 22 with SeO_2_ and *t*BuOOH completed the synthesis of 20.

### 2.4. Chemoenzymatic synthesis of eunicellane and germiane diterpenoids

Eleutherobin (23) is a coral diterpene that possesses the ability to interfere with tubulin polymerization dynamics.^29^ In light of its cytotoxicity, a number of synthetic studies have been performed to prepare eleutherobin and its unnatural derivatives. While these studies have generated valuable structure–activity relationship insights,^30^ they generally employed lengthy sequences with low synthetic efficiency and overall yield. In 2022,^31^ Schmidt and co-workers discovered coral terpene cyclases that produce the general eunicellane skeleton of eleutherobin and cembrene, providing an initial foothold for future synthetic biology approach to produce these diterpenoids. Their effort began with the sequencing and analysis of the metatranscriptome of *E. caribaeorum*, the soft coral producer of eleutherobin. While BLAST analysis did not identify any putative terpene cyclase, the use of hidden Markov model search recognized eight putative terpene cyclases. The authors further observed the colocalization of a terpene cyclase gene (called EcTPS1) with a P450-encoding gene and an acyltransferase-encoding gene in a biosynthetic gene cluster, which is consistent with the likely steps in eleutherobin biosynthesis. To lend support to this hypothesis, EcTPS1 was heterologously expressed in *E. coli* and was found to produce convert GGPP to the known eunicellane^32^ klysimplexin R (24).

**Fig. 3:**
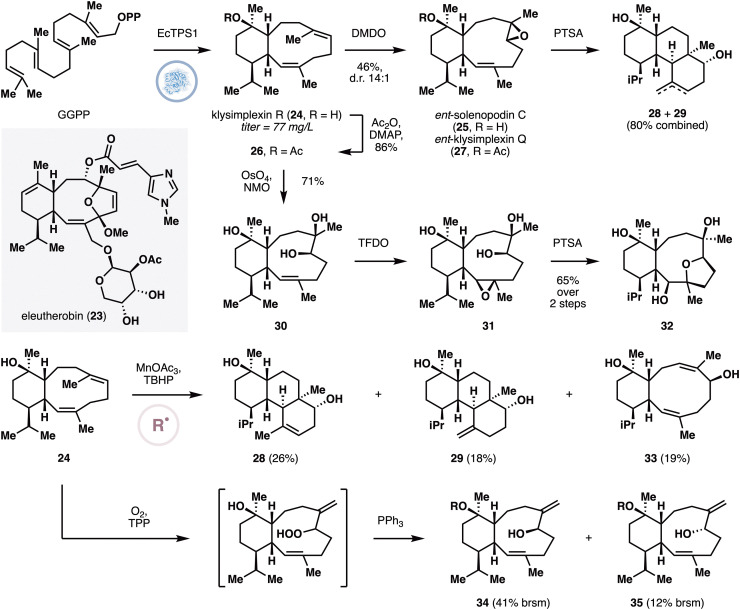
Enzymatic cyclization of GGPP to klysimplexin R and its conversion to oxidized eunicellanes and the germiane skeleton by Scesa and Schmidt.

Scaled-up fermentation of EcTPS1 reaction afforded klysimplexin R (24) with an average titer of 77 mg L^−1^, which was used as a starting point for the divergent synthesis of additional coral diterpenoids (Fig. 3).^33^ The C6–C7 alkene was selectively epoxidized with DMDO to produce a single diastereomer (25), which was found to have identical spectral data but opposite stereoconfiguration to solenopodin C. The same reaction converted 26 to a product (27) that displayed identical spectral data but opposite stereoconfiguration to klysimplexin Q. Under acidic conditions, 25 underwent transannular cyclization to provide two products with the gersemiane skeleton (28 and 29). Alternatively, sequential dihydroxylation of the C6–C7 alkene and TFDO-mediated epoxidation of the C2–C3 alkene afforded epoxide 31, which was treated with PTSA to induce transannular etherification to form 32. Under MHAT conditions,^25^ klysimplexin R could also be transformed to three products. Two of these products have the germiane skeleton, and the last one (33) is a regioisomer of eunicellol A. Finally, 24 was found to undergo ^1^O_2_ ene reaction at the C6–C7 alkene to form two alcohol diastereomers, 34 and 35, after reduction with PPh_3_.

### 2.5. Chemoenzymatic synthesis of (+)-isoagatholactone, (+)-spongian-16-one and 3-deoxychevalone A

**Fig. 4:**
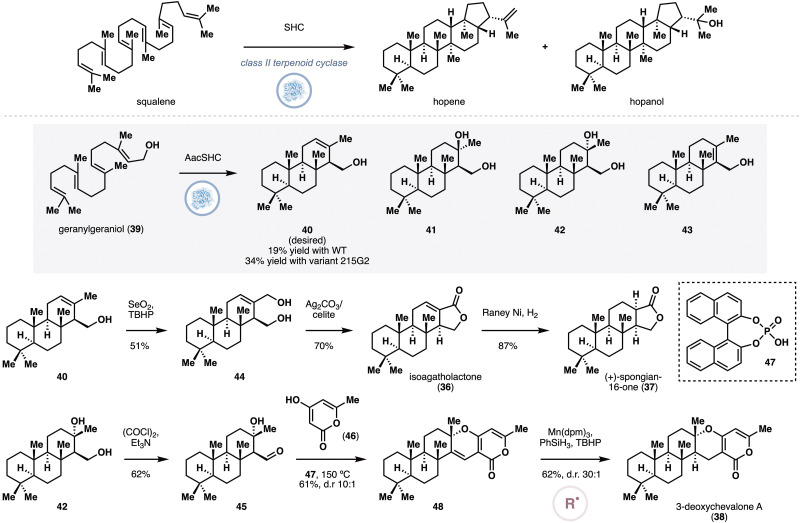
Enzymatic cyclization of geranylgeraniol with engineered *Aac*SHC and conversion of the cyclization products to isoagatholactone, spongian-16-one and 3-deoxychevalone A by Xiang and co-workers.

In contrast to class I terpene cyclases, class II terpene cyclases, use an aspartic acid side chain as a Brønsted acid to protonate alkenes or epoxides to initiate cyclization. Responsible for the conversion of squalene to hopene and hopanol, squalene hopene cyclases (SHC) are among the most-studied class II cyclases.^34^ Over the years, the Hauer group were able to harness this cyclization mode for non-native polyene cyclization through enzymatic Brønsted acid catalysis.^35^ Several engineered variants of an SHC from *Alicyclobacillus acidocaldarius* (*Aac*SHC) are able to accept alkenes, epoxides and carbonyls to form a diverse range of polycyclic ring systems.^36^ More recently, Xiang and co-workers were able to capitalize on the promiscuity of *Aac*SHC in the chemoenzymatic synthesis of (+)-isoagatholactone (36), (+)-spongian-16-one (37) and 3-deoxychevalone A (38, Fig. 4).^37^ The synthesis began with enzymatic polyene cyclization of geranylgeraniol (39) with wild-type *Aac*SHC, which was previously reported to proceed in 12% yield. In the authors’ hands, the desired compound 40 was indeed obtained, but several side products were also detected, namely hydrated products 41 and 42, and the alkene isomer 43. At this stage, the authors performed *in silico* docking to identify several active site residues as candidates for directed evolution, which eventually led to variant 215G2. Containing mutations M132R, A224V and I432T, 215G2 catalyzed the formation of 40 with 34% isolated yield at 0.01 mol% enzyme loading. Allylic oxidation on 40, followed by treatment with Fetizon's reagent produced (+)-isoagatholactone (36), which was converted to (+)-spongian-16-one (37) *via* hydrogenation of 36 in the presence of RANEY® nickel. Finally, 42 was oxidized to the corresponding aldehyde (45), which was coupled with 46 in a formal [3+3] reaction to deliver pyrone 48. Hydrogenation of 48 with Shenvi's conditions^38^ proceeded with excellent chemo- and diastereoselectivity to complete the synthesis of 3-deoxychevalone A (38).

### 2.6. Divergent cross-coupling of albicanol to *ent*-chromozonarol, mycoleptodiscin A and pelorol

**Fig. 5:**
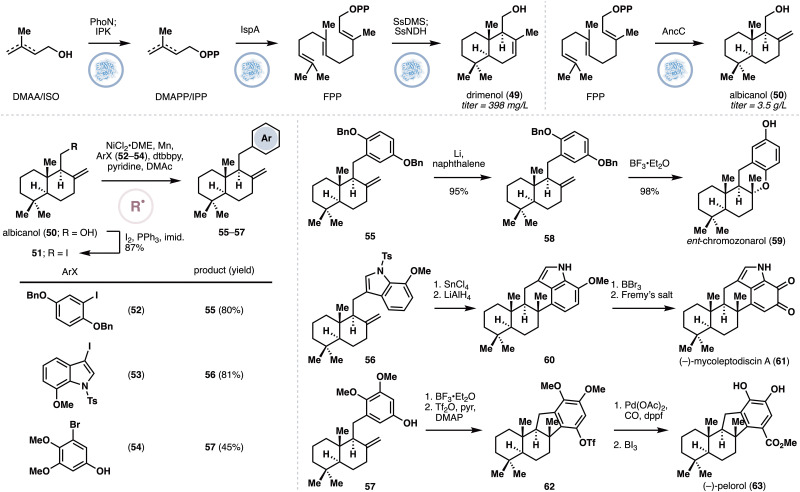
Enzymatic production of drimenol and albicanol and diversification of albicanol to *ent*-chromozonarol, mycoleptodiscin A and pelorol *via* radical cross-couplings by Li, Dong and co-workers.

Earlier this year (2025), the Li and Dong groups reported a collaborative effort to synthesize several meroterpenoids through divergent cross-coupling from simple drimane precursors (Fig. 5), such as drimenol (49) or albicanol (50).^39^ The premise of the approach is to enlist the use of terpene synthases to generate the drimane precursors, which would contain suitable chemical handles for subsequent cross-couplings. Similar to some of the previous case studies, the authors needed to come up with an approach to optimize the titer yield for the drimane precursors. Having previously developed a PhoN-IPK system^40^ to boost the 5-carbon precursor supply for terpene synthases, the authors applied this system in combination with the drimenol synthase SsDMS. Unfortunately, this approach only resulted in a titer yield of 15 mg L^−1^. To address this issue, the authors employed a two-pronged optimization strategy. Firstly, SsDMS was artificially fused with a nudix hydrolase to enhance the hydrolysis of the diphosphate group from the initial terpene cyclization intermediate. This fusion strategy was found to improve the product titer to 68–111 mg L^−1^, depending on the identity of the linker used.^41^ As PhoN catalyzes reversible phosphorylation and hydrolysis reactions on dimethylallylalcohol/isopentenol, a rational engineering approach was pursued to boost the forward phosphorylation reaction under the hypothesis that introducing positive charges in the active site vicinity will improve interactions with the phosphate group. This approach was complemented with an alignment-based mutagenesis and the combined efforts identified three mutations, E122R, T157K and R160K, which led to a titer yield of 398 mg L^−1^. Replacement of SsDMS with the albicanol synthase AncC^42^ allowed for an efficient production of albicanol (50) at a comparable titer. Most notably, a maximum titer of 3.5 g L^−1^ of albicanol could be achieved by conducting the biotransformation in a bioreactor with exogenous feeding of isopentenol. The albicanol so produced was used as a starting point for divergent cross-couplings, specifically with nickel catalysis under Weix's conditions,^43^ which are known to proceed through single electron pathways. Thus, conversion of 50 to the corresponding iodide (51) under Appel conditions, was followed by cross-couplings with the appropriate aryl iodide partners (52–54). Straightforward functional group manipulations completed the chemoenzymatic syntheses of *ent*-chromazonarol (59), mycoleptodiscin A (61) and pelorol (63).

## 3. The use of oxygenases for skeletal functionalization

**Fig. 6:**
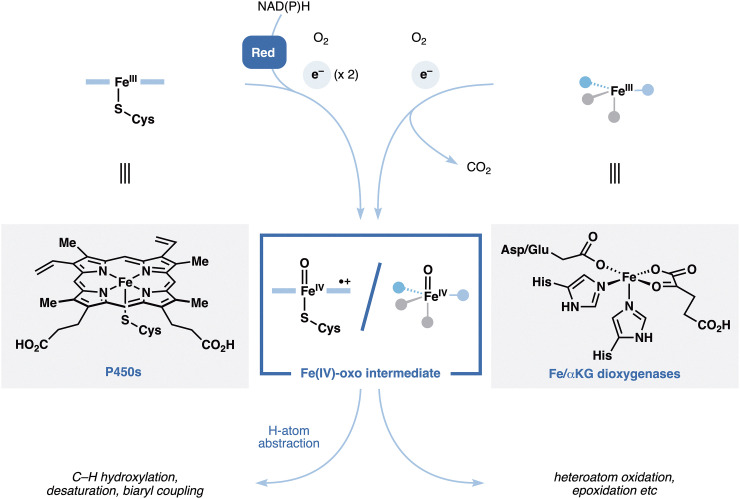
Overview of reaction mechanisms for the P450s and Fe/αKG dioxygenases.

One of the hallmarks of natural product biosynthesis is the involvement of various oxygenases in scaffold tailoring. While the resulting hydroxylation patterns may hold the key for potent biological activities, they also present additional structural complexity and potential chemoselectivity problems for chemical synthesis. Given the wealth of natural oxygenases, a number of research groups have begun to look for enzymatic solutions to address this challenge. While Nature has evolved a wide range of enzyme (super)families for oxidative chemistry, the two major superfamilies that have seen widespread use in organic synthesis are the P450s^44^ and the non-heme iron dioxygenases (NHDs).^45^ Though the two superfamilies use entirely different cofactors and rely on disparate mechanisms for dioxygen activation (Fig. 6), they share one common feature, that is the generation of a highly reactive Fe(iv)–oxo species for oxidative chemistry. This species is capable of performing a wide range of transformations, including C–H hydroxylation, desaturation, biaryl coupling and epoxidation, usually with high levels of site-, stereo- and chemoselectivity, which present a strategic advance in the context of complex molecule synthesis. To date, several strategies have been developed to realize the intended enzymatic oxidations, including transcriptomic analyses to discover new oxygenases, repurposing and engineering of known oxygenases from the native biosynthetic pathways, or screening of known promiscuous oxygenases such as P450BM3 variants.^46^ In turn, the oxidation precursors could be obtained either commercially or through chemical or biological synthesis (*i.e.* heterologous expression). Several research groups have recently taken advantage of the unique features of enzymatic oxidations and use them in combination with modern radical reactions for skeletal construction or modification to achieve efficient chemoenzymatic synthesis of complex natural products.

### 3.1. Chemoenzymatic synthesis of meroterpenoids by combining biocatalytic oxidation and modern radical transformations

Commonly used as fragrance components, sclareolide and sclareol have also been widely used as starting materials in chiral pool synthesis.^47^ particularly towards terpenoid targets that contain a labdane substructure. For example, the Baran group has reported the conversion of sclareolide to ‘borono-sclareolide’, which was used as a divergence point to prepare several phenol-containing meroterpenoids.^48^ Nevertheless, many labdane-containing targets with additional hydroxylation at C3 remain inaccessible with this semisynthetic approach, as chemical methods for direct C–H oxidation would proceed at the undesired C2 position due to stereoelectronic effects.^49^

**Fig. 7:**
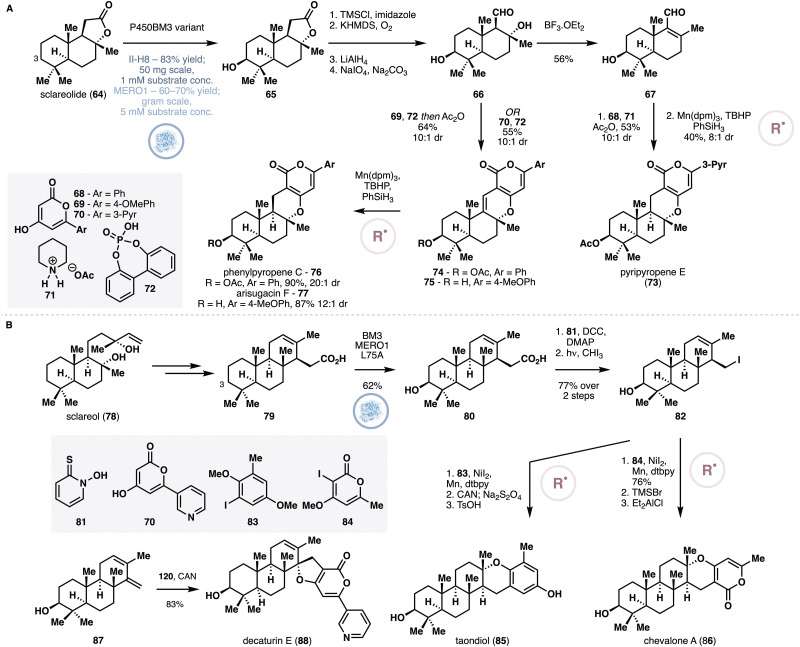
(A) Chemoenzymatic synthesis of α-pyrone meroterpenoids from sclareolide by Renata and co-workers featuring enzymatic oxidation at C3 and MHAT-based alkene reduction. (B) Chemoenzymatic synthesis of taondiol, chevalone A and decaturin E from sclareol by Renata and co-workers featuring enzymatic oxidation of 79 at C3.

An engineered P450BM3 variant, II-H8, was reported by Fasan and co-workers to catalyze a regio- and stereoselective C3 hydroxylation of sclareolide (64) in 83% yield, though the reaction was performed under dilute conditions (50 mg scale *in vitro* reaction with 1 mM substrate concentration).^50^ Using this report as a starting point, the Renata group examined other P450BM3-based catalysts for the same reaction on sclareolide, but ran their screen on a larger scale at higher substrate concentration (5 mM).^51^ A small alanine-scanning library was generated from a P450BM3 variant called 1857, which led to the discovery of a key mutation V328A for improved C3 hydroxylation activity. On gram scale reaction, oxidation of 64 with lysates of *E. coli* expressing 1857 V328A (also called “BM3 MERO1”) provided 60–70% isolated yield of the desired product (65, Fig. 7A). Several routine steps converted 65 to aldehyde 66 or 67, which was coupled with various aromatic fragments in a formal [3+3] annulation (Fig. 5B). Under Shenvi's MHAT conditions,^38^ hydrogenation of the [3+3] adducts proceeded with excellent stereo- and chemoselectivity to complete the synthesis of five different α-pyrone meroterpenoids.

The Renata group further extended this blueprint to the synthesis of several oxidized meroditerpenoids from sclareol (78, Fig. 7B). Firstly, sclareol was converted to acid 79 through a five-step route featuring a radical-based intramolecular Giese addition^52^ and lactone opening. All of the intermediates in this sequence were tested for hydroxylation with the aforementioned alanine-scanning library, which led to the discovery that 79 could be efficiently oxidized at its C3 carbon by variant MERO1 L75A, proceeding with 62% isolated yield on gram scale. The carboxylic acid moiety of 80 was used as a chemical handle to generate further structural complexity and complete the synthesis of four meroditerpenoid targets. Conversion of 80 to the corresponding iodide (82) set the stage for subsequent nickel cross coupling,^53^ which delivered taondiol (85) and chevalone A (86) after routine deprotections. Alternatively, diene 87, obtained *via* elimination of 82, was subjected to a formal [3+2] coupling^54^ to furnish decaturin E (88) and stypodiol.

### 3.2. Chemoenzymatic synthesis of gedunin

**Fig. 8:**
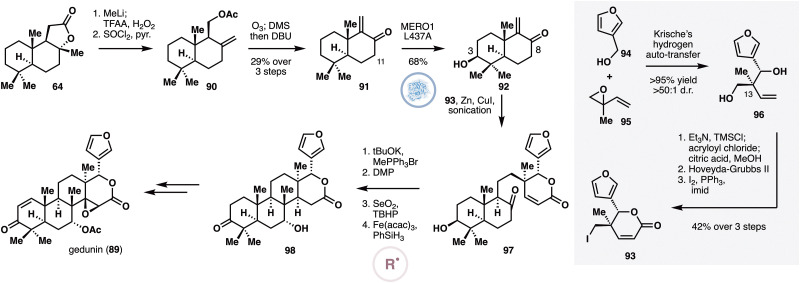
Chemoenzymatic synthesis of gedunin by Renata and co-workers featuring site-selective enzymatic oxidation of 91 with P450BM3 variant MERO1 L437A and intramolecular Giese addition.

Having developed a scalable biocatalytic platform for selective C3 oxidation of sclareolide and related molecules, the Renata group in 2022 reported a convergent chemoenzymatic synthesis of deoxygedunin and gedunin (89),^55^ two limonoids that have been reported to inhibit HSP90 and display antimalarial and neuroprotective properties (Fig. 8). With synthetic modularity in mind, the researchers conceived a fragment coupling with enone 91 for the synthesis, which could be obtained from sclareolide through MeLi addition, Baeyer–Villiger oxidation, C8–OH dehydration, ozonolysis and acetate elimination. Screening of a small panel of P450BM3 library revealed that variant MERO1 L437A was able to oxidize 91 at its C3 carbon with minimal side product arising from alkene epoxidation. Towards iodide 93, furfury alcohol (94) was combined with isoprene monoxide (95) under Krische's hydrogen auto-transfer conditions,^56^ which constructed the key C13 quaternary center with high diastereo- and enantioselectivity. Acrylation of the secondary alcohol was followed by ring-closing metathesis to provide the corresponding unsaturated lactone. Finally, routine conversion of the primary alcohol to iodide generated 93. Fragments 92 and 93 were coupled under Luche's conditions,^57^ and the resulting product was subjected to Wittig olefination with MePPh_3_Br. After DMP oxidation of the C3–OH and allylic oxidation at C7, an MHAT-based Giese coupling^52^ furnished the tetracyclic framework of gedunin. Functional group manipulations consisting of Saegusa oxidation and acetylation at C7 completed the synthesis of deoxygedunin, which was converted to gedunin (89) through epoxidation with *m*CPBA.

### 3.3. Chemoenzymatic synthesis of drimane meroterpenoids *via* enzymatic building block production and enzymatic oxidation

**Fig. 9:**
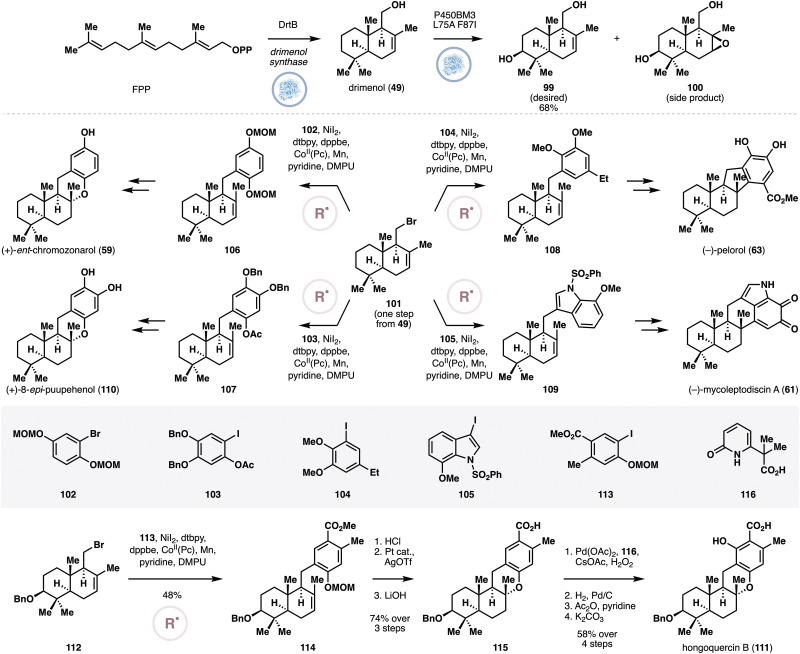
Chemoenzymatic synthesis of drimane meroterpenoids by Xiang and co-workers *via* heterologous production of drimenol, its site-selective oxidation with P450BM3 L75A F87I and radical cross-couplings.

Contemporaneous to the case study outline in Section 2.6, the Xiang group developed a chemoenzymatic approach to drimane meroterpenoids (Fig. 9) that combines heterologous production of drimenol, enzymatic hydroxylation and radical cross-couplings.^58^ Using the authors’ aforementioned system for introducing the MVA pathway in *E. coli*, the product titers of four drimenol synthases^59^ were investigated. Among the four, DrtB demonstrated the highest activity, displaying a titer of 1.5 g L^−1^ for the production of 49. Further metabolic engineering of the pathway improved the titer to 2.1 g L^−1^, providing ample material supply of 49 for the chemoenzymatic synthesis. Oxidation of 49 was initially investigated with P450BM3 F87A, which was capable of forming the desired C3 hydroxylated compound (99) as the main product. Further engineering led to variant P450BM3 L75A F87I, which delivered the desired product in 68% isolated yield on preparative scale. Appel reaction on 49 provided the corresponding primary bromide (101), which was subjected to radical cross-couplings^60^ with a variety of aryl iodide partners. Similar to the approach outlined in Section 2.6, this route eventually completed the synthesis of *ent*-chromazonarol (59), 8-*epi*-puupehenol (110), pelorol (63) and mycoleptodiscin A (61). The same cross-coupling sequence could also be applied for the synthesis of hongoquercin B (111) *via*112, which was prepared from 99 through a four-step sequence. Nickel-mediated cross-coupling of 112 and 113 was followed by MOM deprotection, pyran formation and ester hydrolysis to generate 115. The phenolic alcohol was then introduced *via* palladium-catalyzed C–H oxidation using Yu's protocol.^61^ It should be noted that the authors resorted to this sequence as attempts to effect cross-coupling with the fully-elaborated aryl fragment was unsuccessful. With the general core structure constructed, three additional steps completed the synthesis of hongoquercin B (111).

### 3.4. Divergent chemoenzymatic synthesis of fusicoccane diterpenoids

**Fig. 10:**
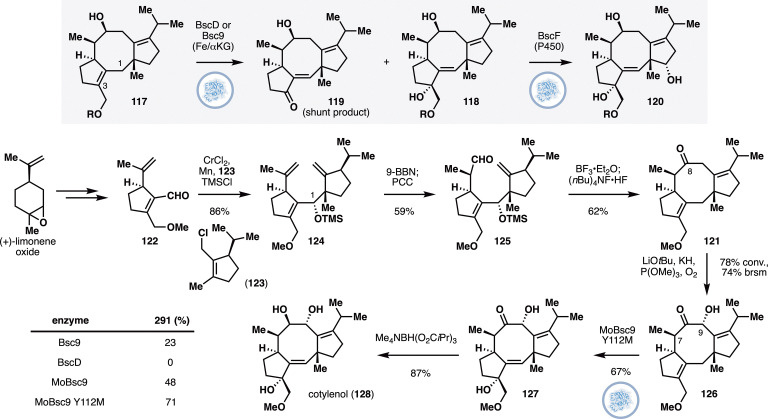
Chemoenzymatic synthesis of cotylenol by Jiang and Renata featuring C3 oxidation of 126 with engineered MoBsc9.

The fusicoccanes are fungal diterpenoids bearing a characteristic 5/8/5-tricyclic ring system.^62^ Within the family, cotylenin A and fusicoccin A are often regarded as the flagship members, as they were one of the firsts to be discovered and were noted to act as modulators of 14-3-3 protein–protein interactions.^63^ More recently, additional family members that contain rearranged tricyclic skeletons have also been isolated, but their biological activities have not been profiled in detail. The Renata group sought to develop a modular chemoenzymatic strategy to target a wide range of family members, including those with rearranged skeletons.^64^ Their design involves the chemical synthesis of a minimally oxidized 5/8/5-tricyclic intermediate, which would then be subjected to a series of chemoenzymatic oxidations. One of the key oxidations in the design hinges on prior biosynthetic studies by Dairi and Oikawa,^65^ which identified the involvement of the NHDs BscD and Bsc9 in the C3 oxidation of compound 117 (Fig. 10). It was known that the reaction is accompanied by the formation of an unwanted shunt product (119) and at the outset, the researchers proposed that this issue could be addressable to additional screening and enzyme engineering.

In a departure from the native biosynthetic pathway, the researchers targeted ketone 121 as their key synthetic intermediate. A fragment coupling strategy was developed to access this ketone featuring the a Nozaki–Hiyama–Kishi coupling^66^ between two cyclopentane units (122 and 123), which in turn were prepared from (+)-limonene oxide and (−)-limonene. A one-pot hydroboration/PCC oxidation delivered aldehyde 125, which was serendipitously found to undergo a Prins cyclization with a transannular hydride shift to provide the desired ketone precursor for subsequent oxidations. Rubottom oxidation of this ketone installed the C9 alcohol and set the stage for subsequent investigation with BscD and Bsc9. While Bsc9 generated the desired oxidation product, significant formation of the undesired shunt product (*cf.* compound 119) was also observed. To address this issue, the researchers performed Genome Neighborhood Network analysis^67^ to pick several homologs of Bsc9 for further reaction with 126. Among the homologs, MoBsc9 from *M. oryzae* offered improved reaction conversion and superior product distribution. Further active site engineering of MoBsc9 identified mutation Y112M that further improved the reaction conversion and product ratio, allowing the desired product (127) to be obtained in 67% isolated yield on 30 mg scale reaction. Finally, the conversion of 127 to cotylenol (128) was conducted following a protocol previously developed by Nakada and co-workers.^68^

**Fig. 11:**
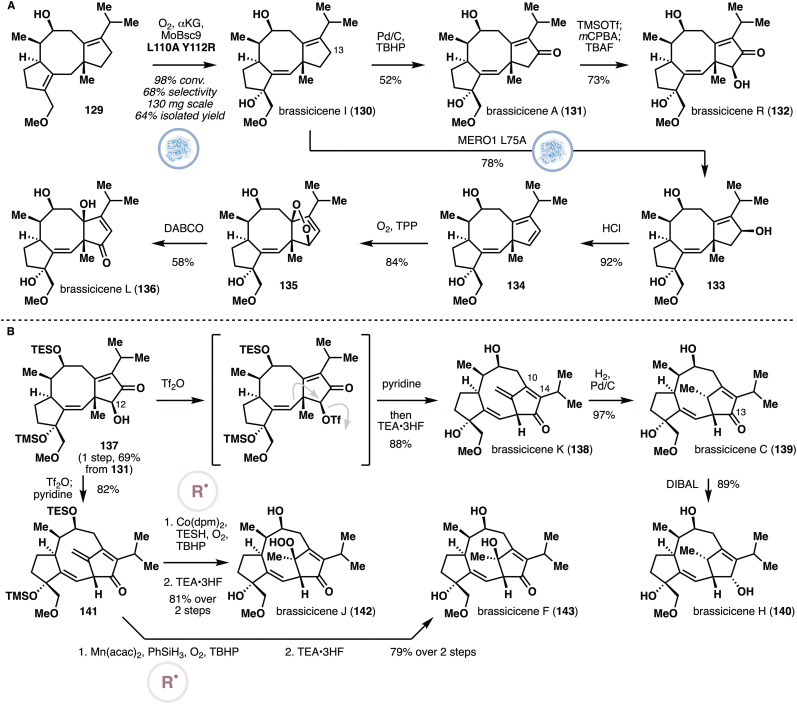
Chemoenzymatic synthesis of oxidized and rearranged fusicoccanes by Jiang and Renata. (A) Synthesis of brassicicenes A, I, L and R. (B) Synthesis of brassicicene C, F, J, and H.

Through a similar directed evolution campaign, mutations L110A and Y112R on MoBsc9 were discovered to improve the chemoselectivity of reaction with alcohol 129 (Fig. 11), delivering brassicicene I (130) in 64% isolated yield on 130 mg scale. Brassicicene I was further found to undergo selective C13 hydroxylation with P450BM3 variant MERO1 L75A in 78% yield. On the other hand, palladium-catalyzed allylic oxidation^69^ formed the enone product, completing the synthesis of brassicicene A (131), which was converted to brassicicene R (132) through routine Rubottom oxidation. The allylic alcohol from MERO1 L75A oxidation could be dehydrated to the resulting cyclopentadiene (134), which was then subjected to ^1^O_2_ Diels–Alder and Kornblum–Delamare rearrangement to afford brassicicene L (136). Towards the rearranged fusicoccanes, a partially protected derivative of brassicicene R (137) was submitted to a chemoselective Wagner–Meerwein shift in the presence of Tf_2_O and pyridine. After silyl ether deprotection, brassicicene K (138) was obtained.^70^ Selective hydrogenation of the *exo* methylene of 138 generated brassicicene C (139), which was reduced with DIBAL reduction to afford brassicicene H (140). Alternatively, 141 could also be subjected to Mukaiyama hydroperoxidation^71^ and deprotection to deliver brassicicene J (142), or Mukaiyama hydration and deprotection to provide brassicicene F (143). In total ten fusiccocane natural products were prepared using this chemoenzymatic approach.

### 3.5. Chemoenzymatic synthesis of non-terpenoid targets

#### 3.5.1. Renata's synthesis of podophyllotoxin and related aryltetralin lignans

**Fig. 12:**
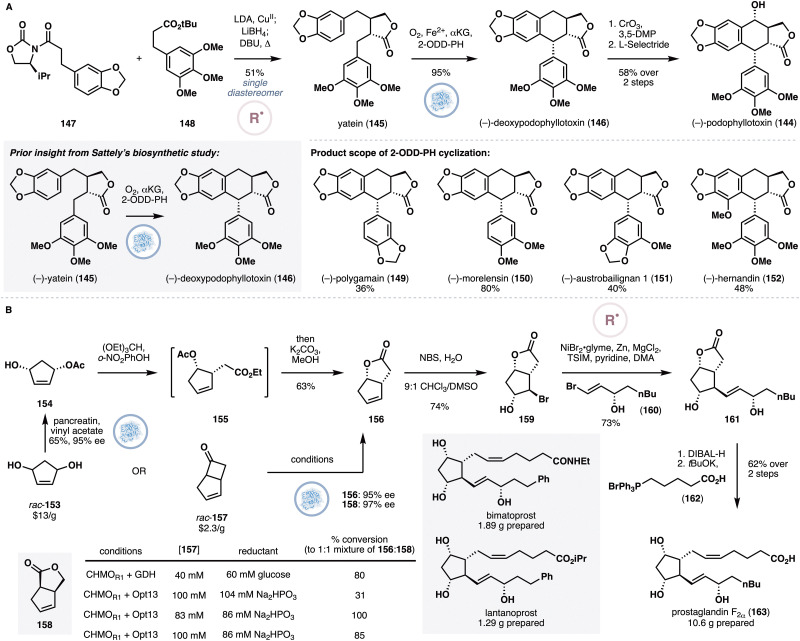
(A) Chemoenzymatic synthesis of podophyllotoxin by Renata and co-workers *via* oxidative enolate coupling and enzymatic oxidative cyclization with 2-ODD-PH. (B) Chemoenzymatic synthesis of prostaglandins by Li and co-workers.

Podophyllotoxin (144) is an aryltetralin lignan that can serve as a highly potent microtubule depolymerizing agent.^72^ This property has led to semisynthetic efforts to optimize the anticancer properties of the molecule, culminating in the invention of two derivatives, etoposide and teniposide, as chemotherapy agents. In 2019,^73^ the Renata group reported an asymmetric chemoenzymatic approach to podophyllotoxin and additional aryltetralin lignans by drawing inspiration from a prior biosynthetic study by Lau and Sattely (Fig. 12A),^74^ which suggested the involvement of a late-stage oxidative cyclization from yatein (145) by an NHD, 2-ODD-PH, in the biogenesis of podophyllotoxin. The general blueprint of their approach is to develop a concise chemical access to yatein, and subject it to chemoenzymatic redox adjustments to arrive at podophyllotoxin. Towards yatein, the authors enlisted the use of oxidative enolate coupling methodology developed by Baran,^75^ which presumably proceeds through single electron oxidation, followed by radical–radical coupling. Thus, 147 and 148 were enolized with LDA and treated *in situ* with a Cu^II^ salt to furnish the desired dicarbonyl product. Selective monoreduction with LiBH_4_ and equilibration of the initial diastereomeric mixture with DBU generated yatein as a single diastereomer in 51% overall yield. Oxidative cyclization in the presence of 2-ODD-PH generated the desired tetracycle in 95% yield. Finally, benzylic oxidation of 146 with CrO_3_ and 3,5-dimethylpyrazole, followed by ketone reduction with l-selectride completed the synthesis of podophyllotoxin. In this work, the authors also conducted a systematic substrate–activity relationship profiling of 2-ODD-PH, which resulted in the synthesis of five additional aryltetralin lignans, including the natural products polygamain (149), morelensin (150), austrobailignan 1 (151) and hernandin (152). It should be noted that the Fuchs and Kroutil group^76^ contemporaneously reported a complementary approach to podophyllotoxin by combining a biocatalytic kinetic resolution of a dibenzylbutyrolactone precursor and oxidative cyclization with 2-ODD-PH.

#### 3.5.2. Li's synthesis of prostaglandins

Prostaglandins are a group of lipid-derived, hormone-like substances that play key regulatory roles in various metabolic functions.^77^ In light of this property, there has been a lot of interests in creating prostaglandin derivatives with improved chemical stability and reduced side effects. To date, more than 20 prostaglandin-derived drugs have been clinically approved and numerous *de novo* syntheses have also been reported. A well-known strategy in these syntheses involves the use of Corey lactone,^78^ which contains a primary alcohol for a variety of derivatizations through two-electron chemistry. The Li group sought to complement this approach by developing a bromide counterpart of the lactone, which would be amenable to use in radical cross-couplings.^79^

Toward the above goal, the authors explored two sequences to prepare the target bicycle (Fig. 12B). Firstly, *meso*-diol 153 was desymmetrized through lipase-mediated esterification,^80^ and then subjected to a Johnson–Claisen rearrangement and lactonization. This sequence was eventually abandoned in favor of an alternative one that commenced from a cheaper starting material, cyclobutanone 157. Enzymatic Baeyer–Villiger^81^ of 157 in the presence of CHMO_rhodo1_ and Opt13 for NADPH regeneration afforded lactones 156 and 158 in 95% and 97% ee respectively. Remarkably, this reaction could be conducted at 83 mM substrate concentration, affording >100 g of 156 throughout the synthetic campaign. After extensive optimization, 156 could be converted to the desired bromohydrin diastereomer 159, which set the stage for the key radical cross-coupling. Initial foray into this reaction led to significant formation of an undesired epoxide byproduct, in addition to the desired product 161. This issue was solved by the addition of trimethylsilylimidazole for *in situ* alcohol protection. With this modification, the desired cross-coupling proceeded smoothly either with one equivalent of pyridine or 0.15 equivalent of a bidentate bipyridine ligand. A range of vinyl and aryl bromides were found to be suitable coupling partners, affording at least 60% yield. Starting from cross-coupling product 161, two uneventful steps, namely lactone reduction and aldehyde homologation, led to prostaglandin F_2α_ (163). Using a similar sequence, additional prostaglandins, such as bimatoprost, fluprostenol, cloprostenol and lantanoprost, were prepared. As a testament to the robustness of the route, each of these molecules was obtained in more than 1 g quantity.

#### 3.5.3. Chemoenzymatic functionalization of piperidine carboxylic acid isomers

Piperidine is one of the most common heterocycles found in clinically-approved drugs and pharmaceutical leads. Especially valuable in this area are chiral piperidines that contain multiple substitutions. A recent survey suggests that di- and trisubstituted piperidines cumulatively make up for approximately 71% of piperidine-based drugs.^82^ The ubiquity of multisubstituted piperidines has spurred the development of various methods for their construction. Common approaches^83^ in this area include *de novo* ring synthesis *via* intramolecular cyclization and dearomative functionalization of pyridines. However, *de novo* ring construction often requires lengthy substrate preparation and asymmetric dearomative functionalization still requires chiral auxiliary and is often beset by regioselectivity challenges. Thus, there is still an unmet demand for an efficient synthesis of densely-functionalized, chiral piperidines with diverse substitution patterns.

**Fig. 13:**
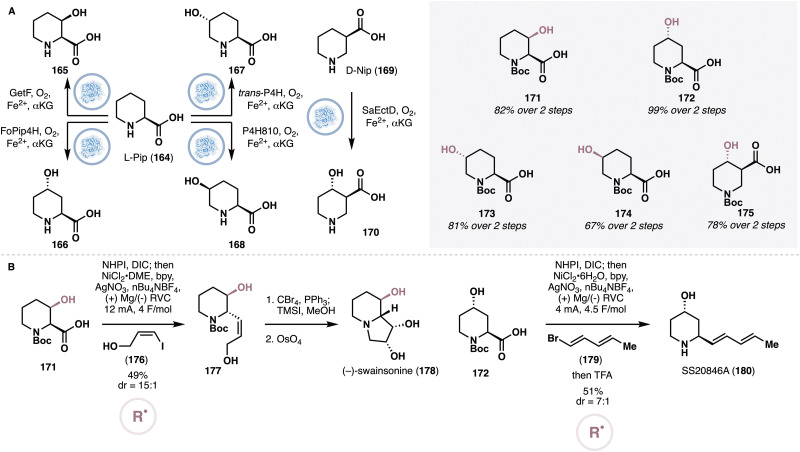
(A) Divergent enzymatic oxidation of l-pipecolic acid to 165–168 and enzymatic oxidation of d-nipecotic acid to 170. (B) Baran and Renata's approach to swainsonine and SS20846A *via* electrochemical cross-couplings of 171 and 172 respectively.

A recent collaborative work^84^ from the Baran and Renata groups developed a chemoenzymatic approach to complex piperidines by relying on site-selective oxidations of several piperidine carboxylic acid regioisomers, followed by radical cross-couplings for rapid elaboration. The researchers noted several early reports on oxidizing l-pipecolic acid (l-Pip, 164, Fig. 13A) at its C2, C3 and C4 positions with the NHDs GetF (C2),^85^ FoPip4H (C3)^86^ and *trans*-P4H/P4H810 (*trans*/*cis* C4)^87^ respectively and embarked on an extensive screening campaign to systematically optimize the performance of the enzymes for large-scale biotransformations. Eventually, a set of conditions was identified for each enzyme that allows gram-scale access to building blocks 165–168 on gram scale. In addition, the researchers also sought to identify a biocatalytic system for the oxidation of nipecotic acid (d-Nip, 169). Screening of a small pool of NHDs identified ectoine 5-hydroxylase^88^ from *S. alaskensis* as a viable biocatalyst for the C4 hydroxylation of d-Nip, which was further optimized through evaluation of various reaction parameters to eventually afford 100% conversion on 15 mM substrate loading. Cumulatively, five key fragments (171–175) were successfully prepared in gram or near-gram quantities from the enzymatic oxidations after simple Boc protection.

Starting from 171–175, a number of synthetic targets, including alkaloidal natural products and active pharmaceutical ingredients, were successfully obtained through the use of Ni-based cross couplings that rely either on the free carboxylic acid or the newly-introduced alcohol as cross-coupling handle. To specifically highlight in the context of natural product synthesis (Fig. 13B), 171, obtained *via* biocatalytic oxidation with GetF, was first converted to its NHPI ester counterpart and submitted to an electrochemical decarboxylative cross-coupling^89^ with vinyl iodide 176 to furnish allyl alcohol 177 in 49% yield and 19 : 1 dr. The primary alcohol of 177 was selectively converted to the corresponding bromide under standard Appel conditions and removal of the Boc group led to simultaneous cyclization to afford a penultimate bicyclic intermediate. Finally, alkene dihydroxylation in the presence of OsO_4_ completed the synthesis of (−)-swainsonine (178). Conversely, 172, obtained *via* biocatalytic oxidation with FoPip4H, was combined with vinyl bromide 179 under the same electrochemical decarboxylative cross-coupling conditions. Following Boc group removal with TFA, the *Streptomyces* alkaloid SS20846A (180) was obtained.

#### 3.5.4. Baran's synthesis of saxitoxin

**Fig. 14:**
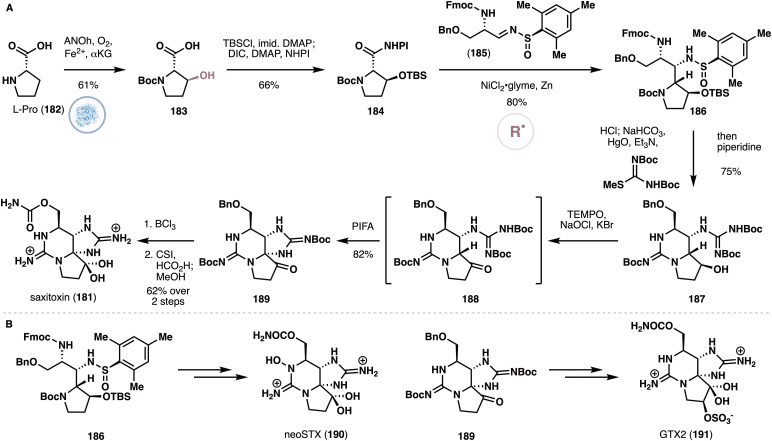
(A) Baran's synthesis of saxitoxin featuring enzymatic oxidation of l-proline with ANOh and radical coupling with sulfinimine 185. (B) Extension of the approach to the synthesis of neosaxitoxin and gonyautoxin 2.

Saxitoxin (181) is a well-known parasitic shellfish toxin that exhibits lethal neurotoxicity by inhibiting voltage-gated sodium channels.^90^ Its unusual structure, in combination with its potential use as anesthetics in pain treatment and management, has led to the development of many synthetic approaches to the natural product. Very recently, the Baran group, in collaboration with scientists from Merck, reported^91^ a concise chemoenzymatic access to 181 and several congeners that features the use of radical cross-coupling and biocatalytic oxidation as its key steps. The researchers posited that the substituted pyrrolidine motif on 181 could potentially arise from a hydroxylated derivative of l-proline (182). However, additional stereochemical considerations suggested the need for *trans*-3-hydroxyproline as starting material. Due to its prohibitive cost, a variety of approaches were investigated for its large-scale synthesis, culminating in the development of a biocatalytic oxidation route. While oxidation of l-proline to its *trans*-3-hydroxy counterpart has never been reported previously, the team found that a previously uncharacterized fungal NHD named ANOh was able to generate a 1.2 : 1.0 mixture of the *trans*-3 and *trans*-4 isomers from l-proline (Fig. 14). Further engineering of this enzyme resulted in a variant called CDX-090, which exclusively produces *trans*-3-hydroxyproline with exceptionally high reaction titer (substrate loading of >30 g L^−1^).

Following Boc and TBS protection, the resulting intermediate was submitted to a decarboxylative coupling^92^ with sulfinimine 185 to provide 186 as a single diastereomer in 80% yield. The team noted that the more conventional Mannich disconnection was also attempted but was met with no success. A telescoped process was next developed featuring Boc deprotection, double guanidylation and intramolecular ring closure to generate bicycle 187 in 75% yield. After oxidation under Anelli conditions, the resulting ketone was immediately treated with PIFA *in situ* to effect an oxidative C–N bond formation a to the ketone, thereby forging the signature tricyclic ring system of 181. Here, the researchers also examined alternative C–H amination or oxidation conditions for forge the C4–N9 bond, but all these conditions did not lead to any promising results. To complete the synthesis of 181, global deprotection with BCl_3_ and carbamoylation of the primary alcohol were performed, resulting in a seven-step synthesis of 181 (longest linear sequence). Using the initial synthesis of 181 as a template, the authors further showed that intermediates 186 and 189 could be used as starting points for accessing a number of congeners (Fig. 14B), including neosaxitoxin (neoSTX, 190) and gonyautoxin 2 (GTX2, 191).

## 4. Conclusions

This review outlines numerous case studies wherein enzymatic and radical reactions were combined in a strategic fashion to efficiently synthesize complex natural products. For example, the use of terpene cyclases enables the formation of multiple C–C bonds and unique ring systems from simple, acyclic precursors in one step. In contrast, chemical synthesis of the analogous carbocycles usually requires multi-step sequences that also need to take into account various stereoelectronic and thermodynamic factors. When combined with the unique reactivity profile and the high functional group compatibility of modern radical reactions, it is perhaps not a surprise that the resulting hybrid approach would result in unparalleled synthetic efficiency. Similar benefits in synthetic economy can also be obtained by combining modern radical reactions for skeletal construction and enzymatic tailoring with oxygenases as modern radical reactions offer the ability to make unconventional bond disconnections and oxygenases are capable of performing selective oxidations and functionalizations that are otherwise challenging to perform with traditional chemical methods. Despite these advantages, several challenges—especially in enzymatic reaction development—still exist and need to be addressed to further expand the generality of the chemoenzymatic strategies outlined above. Not all terpene cyclases are capable of producing the desired carbocyclic scaffolds in high titers and extensive metabolic and protein engineering may be needed to boost the titers to levels that are appropriate for material supply in multi-step syntheses. It is also generally difficult to predict the substrate promiscuity of a given enzyme at present and in the context of enzymatic oxidation specifically, we still do not have sufficient capability to predict the site-selectivity of a given oxygenase when applied on substrates whose structure differ substantially from what have been reported previously. As such, enzymatic oxidations on such scaffolds may require extensive screening for the identification of the right enzyme(s). Presently, such screening can be done with “brute force” chromatographic methods, but incorporation of more modern platforms such as droplet microfluidic^93^ for higher screening throughput, or deep learning models for activity prediction,^94^ could alleviate the screening burden. As can be seen in the case studies presented, there has been a lot of emphasis on the use of terpene cyclases and oxygenases in current approaches in chemoenzymatic synthesis of natural products. However, this is not to say that other enzyme families are less applicable. In fact, there is a lot of untapped opportunities in the use of other enzyme families in this author's view. The recent surge in the development of new-to-nature biocatalytic reactions, especially in the area of radical reactions,^95^ is particularly exciting in this regard, as they open up a gamut of possibilities for even more inventive reaction combinations in chemoenzymatic total synthesis. Progress in chemoenzymatic synthesis has also been slowed by limited avenues for knowledge dissemination and accessibility to resources—for example: a lack of searchable database for biocatalytic reactions and a lack of familiarity with the general concepts and the technical aspects of biocatalysis among practitioners of organic chemistry—, and more concerted educational efforts,^96^ including the introduction of biocatalysis/enzyme catalysis in formal curricula and the development of biocatalysis-focused workshops, could help accelerate progress. These challenges are by no means insurmountable and on the whole, the increasing number of chemoenzymatic total synthesis that features a combination of enzymatic and radical reactions in the past decade heralds a rosy long-term prospect. Thus, we anticipate that the field will see exponential advancements in the future in accord with new technological advances in both areas.

## Conflicts of interest

There are no conflicts to declare.

## Data Availability

No primary research results have been included and no new data were generated or analyzed as part of this review.
